# Human papillomavirus types in invasive cervical cancer worldwide: a meta-analysis

**DOI:** 10.1038/sj.bjc.6600688

**Published:** 2003-01-28

**Authors:** G M Clifford, J S Smith, M Plummer, N Muñoz, S Franceschi

**Affiliations:** ^1^International Agency for Research on Cancer, 150, cours Albert Thomas, 69008 Lyon, France

**Keywords:** human papillomavirus, cervical carcinoma, squamous cell carcinoma, adenocarcinoma, epidemiology, literature review

## Abstract

This study investigated regional variations in the contribution made by different human papilloma (HPV) types to invasive cervical cancer (ICC). A total of 85 studies using polymerase chain reaction to estimate HPV prevalence in ICC were identified. Data on HPV prevalence were extracted separately for squamous cell carcinoma (SCC) and for adeno- and adenosquamous-carcinoma (ADC). A total of 10 058 cases (8550 SCC, 1508 ADC) were included in pooled analyses. The most common HPV types in ICC were, in order of decreasing prevalence, HPV16, 18, 45, 31, 33, 58, 52, 35, 59, 56, 6, 51, 68, 39, 82, 73, 66 and 70. In SCC, HPV16 was the predominant type (46–63%) followed by HPV18 (10–14%), 45 (2–8%), 31 (2–7%) and 33 (3–5%) in all regions except Asia, where HPV types 58 (6%) and 52 (4%) were more frequently identified. In ADC, HPV prevalence was significantly lower (76.4%) than in SCC (87.3%), and HPV18 was the predominant type in every region (37–41%), followed by 16 (26–36%) and 45 (5–7%). The overall detection of HPV DNA was similar in different regions (83–89%). A majority of ICC was associated with HPV16 or 18 in all regions, but approximately a quarter of all ICC cases were associated with one of 16 other HPV types, their distribution varying by region.

Epidemiological studies have clearly established human papilloma-virus (HPV) infection as the central cause of invasive cervical cancer (ICC). This is the second most common cancer among women worldwide and the most common female cancer in large areas of the developing world where an estimated 80% of new cases arise (Parkin *et al*, 1999). Studies in 22 countries, coordinated by the International Agency for Research on Cancer (IARC), identified HPV DNA in almost all (99.7%) (of about 1000) cases of cervical cancer (Walboomers *et al*, 1999).

Approximately 40 distinct HPV types are known to infect the genital tract and epidemiological studies to date suggest that at least 14 of these, called oncogenic or high-risk (HR) types, are significantly associated with progression to ICC (Bosch *et al*, 1995). Most of these HR types are phylogenetically related to either HPV16 (31, 33, 35, 52 and 58) or HPV18 (39, 45, 59 and 68) (Chan *et al*, 1995). Limited evidence suggests that their distribution may vary by region (Bosch *et al*, 1995).

HPV vaccines hold great promise to reduce the global burden of ICC any potential vaccine be multivalent since prior infection with one type does not appear to decrease the risk of infection by another HPV type (Koutsky *et al*, 2002; Combita *et al*, 2002; Liaw *et al*, 2001). In this however, to collate all relevant published data to identify the most prevalent HPV types associated with ICC worldwide and within five geographic regions.

## Materials and methods

### Study selection

Source material was selected from citations listed in *Medline* and *ISI Current Contents* databases and from references cited in the selected papers. Key search terms included: cervical cancer, HPV, human, female, and polymerase chain reaction (PCR). The review was limited to studies that included a minimum of 20 ICC cases; carcinomas *in situ* were excluded. Studies had to provide a clear description of the use of PCR-based assays to identify HPV DNA. Studies using nonamplified hybridisation methods only were excluded based on the reduced sensitivity of such methods in comparison to PCR (Gravitt *et al*, 1991; Schiffman *et al*, 1991; Guerrero *et al*, 1992). Furthermore, articles were only included if type-specific prevalence of at least one HPV type other than HPV6, 11, 16 or 18 was reported. For articles where study methods suggested that additional type-specific data were available, these data were requested from the authors (Yang *et al*, 1997; Eluf-Neto *et al*, 1994; Chaouki *et al*, 1998; Meyer *et al*, 1998; Chen *et al*, 1999; Lin *et al*, 2001). If data or data subsets had been published in more than one article, only the publication with the largest sample size was included.

### Data abstraction

For each study, the following key information was extracted: country of sample; sample size; mean age; study year; distribution of cases by histological type; type of cervical specimen (e.g., fresh/fixed biopsies or exfoliated cells) and PCR primers used to detect HPV positive samples; type-specific and overall prevalence of HPV infection. Where available, data on HPV-specific prevalence were extracted independently for squamous cell carcinoma (SCC) and for adeno- and adenosquamous carcinoma (henceforth collectively termed ADC). Where histology-specific HPV prevalence was not reported, cases were classified as being of ‘unspecified’ histology. Each study was classified into one of five geographical regions: Africa, Asia, Europe, North America and Australia, or South and Central America. For studies comparing HPV prevalence across regions (Munoz *et al*, 1992; Bosch *et al*, 1995; Sebbelov *et al*, 2000), data were separated into their regional components.

### Studies included

Of studies published up to February 2002 on *Medline* identified by our search criteria, 82 qualified for inclusion (no additional studies were included from *ISI Current Contents*). Three studies were conference abstracts containing the detailed information required for inclusion (Illades-Aguiar *et al*, 2000; Nindl *et al*, 2001; Rabelo-Santos *et al*, 2001). In the course of contacting authors, additional data became available for two studies expanded since the original publication (Burger *et al*, 1996; Andersson *et al*, 2001). Detailed information on the design of each of the 85 included studies is listed in the appendix.

### Estimation of type-specific prevalence

HPV prevalence data were expressed as percentages of all cases tested for HPV. Multiple infections (3.7% of all ICC cases) were separated into constituent types, thus type-specific prevalence represents that in either single or multiple infections. Cases with specimens considered to be inadequate for PCR testing were excluded. Type-specific prevalence is presented for the 18 most common HPV types as identified by this review (HPV types 6, 16, 18, 31, 33, 35, 39, 45, 51, 52, 56, 58, 59, 66, 68, 70, 73 and 82 also known as MM4, W13B or IS39) in order of descending prevalence for each subgroup analysis. Consensus PCR primers My09/11 (Bernard *et al*, 1994), GP5^+^/6^+^ (Chaouki *et al*, 1998) and SPF10 (Kleter *et al*, 1999) were considered to amplify all 18 HPV types, L1C1/L1C2 (Nakagawa *et al*, 1996) to amplify all types, but HPV73 and 82, GP5/6 to amplify types 6, 11, 16, 18, 31, 33, 35 and 45 only (Roda Husman *et al*, 1995), and pU1M/2R (Harima *et al*, 2002) to amplify types 6, 16, 18, 31, 33, 35, 52, 56, 58 and 59 only. For other consensus and type-specific PCR primers, only those HPV types specified in the individual reports were considered amplifiable. For HPV-specific prevalence, only studies testing for a particular HPV type contribute to the analysis for that type, and therefore sample size varies between the type-specific analyses.

### Statistical analyses

Sources of variation in overall HPV prevalence were investigated by unconditional multiple logistic regression analysis (Breslow and Day, 1980). The final model included the following sources of variation: geographical region, histological type of ICC, type of specimen for HPV DNA testing, and type of PCR primers used. Mean age and study year were found not to be significantly related to overall HPV prevalence. Adjustment of overall HPV prevalence for these variables was done using the *adjust* command in Stata version 7.0, based on probability estimates from the logistic regression model. Confidence intervals for overall HPV prevalence were calculated assuming the nonindependence of cases within the same study using the *cluster* option in Stata (White, 1980). *P*-values comparing the prevalence of particular HPV types in subsets of ICC cases refer to *χ*^2^ tests.

## Results

### Meta-analysis of overall HPV prevalence

A total of 10 058 ICC cases from the 85 identified studies were included in this meta-analysis of HPV prevalence (Table 1

**Table 1:** Region- and histology-specific distribution of included studies and ICC cases

				**Number (%) of cases with histology-specific HPV data**		
**Region**	**No. of studies**	**Countries represented**	**No. of cases**	**SCC**	**ADC**	**Unspecified**
Africa	6	Algeria, Benin, Guinea, Mali, Morocco, Senegal, South Africa, Tanzania, Uganda	609	204 (33.5)	21 (3.4)	384 (63.1)
Asia	28	Mainland China, India, Indonesia, Japan, Korea, Malaysia, Philippines, Taiwan, Thailand	3091	2273 (73.5)	381 (12.3)	437 (14.1)
Europe	32	Austria, Czech Republic, Denmark, Finland, France, Germany, Greece, Greenland, Holland, Hungary, Ireland, Italy, Norway, Poland, Russia, Sweden, UK	3336	2010 (60.3)	603 (18.1)	723 (21.7)
North America and Australia	13	Australia, Canada, USA	1562	914 (58.5)	450 (28.8)	198 (12.7)
South and Central America	12	Argentina, Bolivia, Brazil, Chile, Colombia, Costa Rica, Cuba, Honduras, Mexico, Panama, Paraguay, Peru	1460	424 (29.0)	53 (3.6)	983 (67.3)
Total	85		10 058	5825 (57.9)	1508 (15.0)	2725 (27.1)

HPV=human papillomavirus; ICC=invasive cervical cancer; SCC=squamous cell carcinoma; ADC=adeno/adenosquamous carcinoma.

). A majority of cases came from studies performed in Asia (31%) and Europe (33%), with African studies representing the smallest proportion of cases (6%). HPV prevalence was reported stratified by histological type for 73% of the cases: 5825 SCC cases and 1508 ADC cases. In total, 12 studies included only SCC and seven studies included only ADC.

Adjusted overall HPV prevalence ranged from 79.3% in Asia to 88.1% in North America and Australia, but did not differ significantly between regions (Table 2

**Table 2:** Prevalence of HPV by region, histological type, HPV DNA specimen and PCR primers used

**Variable**	**No. of studies**	**No. of cases**	**Crude HPV prevalence (%)**	**Adjusted^a^ HPV prevalence (%)**	**95% confidence intervals**
*Region*					
Africa	6	609	88.8	86.5	(76.4–92.7)
Asia	28	3091	83.1	79.3	(73.7–84.0)
Europe	32	3336	85.9	86.7	(82.5–90.0)
North America and Australia	13	1562	87.5	88.1	(83.6–91.5)
South and Central America	12	1460	89.3	87.7	(83.1–91.2)
					
*Histological type*					
Squamous cell carcinoma	47	5825	86.9	87.3	(84.8–89.5)
Adeno(squamous) carcinoma	45	1508	76.7	76.5	(72.3–80.3)
Unspecified	48	2725	89.0	89.2	(85.1–92.3)
					
*HPV DNA specimen*					
Fixed biopsies	34	3324	84.3	83.3	(78.9–86.9)
Fresh biopsies	27	3992	88.4	87.8	(84.9–90.2)
Unspecified biopsies	8	496	87.1	87.2	(82.3–90.9)
Cervical exfoliated cells	13	1444	80.1	78.9	(68.5–86.5)
Cells and biopsies	3	802	93.5	92.5	(87.3–95.7)
					
*Primers*					
MY09/11	31	4355	85.9	83.3	(80.1–86.0)
GP5/6	6	506	80.8	77.8	(64.9–87.0)
GP5+/6+	14	1681	92.2	90.1	(85.0–93.6)
SPF10	3	275	96.7	97.2	(87.9–99.4)
PU1M/2R	6	376	80.9	79.4	(68.8–87.0)
L1C1/C2	5	655	91.2	88.0	(77.2–94.1)
Combination	9	1351	88.4	86.4	(77.9–92.0)
Other	4	166	84.3	89.3	(75.2–95.9)
TS-PCR only	7	693	73.6	74.7	(63.8–83.2)

a Adjusted for histological type, region, HPV DNA specimen and PCR primers.

). HPV DNA was significantly less likely to be detected in ADC (76.5%) than in SCC (87.3%) (*P*<0.001). DNA detection in ICC of unspecified histology (89.2%) was similar to that in SCC.

Adjusted HPV prevalence was significantly higher from studies testing both cells and biopsies for HPV DNA (92.5%) than from studies testing either cervical exfoliated cells (78.9%) or fixed biopsies (83.3%) only. For PCR primers, highest HPV prevalence was obtained in studies using SPF10 primers (97.2%) and the lowest in studies using type-specific PCR (TS-PCR) only (74.7%). Adjusted overall HPV prevalence varied between 77.8 and 90.1% for other primer sets, but these differences were not statistically significant.

### Meta-analysis of HPV type-specific prevalence

**Figure 1:**
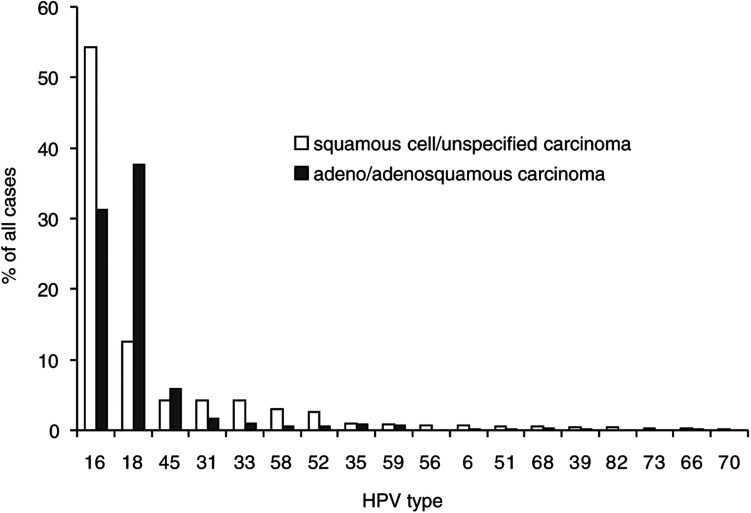
Type-specific prevalence of HPV in 10 058 worldwide cases of invasive cervical cancer by histological type.

Owing to their similar overall and type-specific HPV prevalence, ICC of unspecified histology were combined with SCC for comparison of HPV type-specific prevalence by histological type (Figure 1

). The most common HPV types identified were, in order of decreasing prevalence, HPV16, 18, 45, 31, 33, 58, 52, 35, 59, 56, 6, 51, 68, 39, 82, 73, 66 and 70. Other HPV types were detected in no more than 0.2% of ICC cases. There was considerable variation in HPV-specific prevalence between SCC and ADC. HPV16 was identified more often in SCC (55.2%) than in ADC (31.3%) (*P*<0.001). The same was found for the HPV16 phylogenetically related types 31, 33, 52 and 58 (*P*<0.001), but not 35. Conversely, HPV18 was more prevalent in ADC (37.7%) than in SCC (12.3%) (*P*<0.001). The HPV18 phylogenetically related type 45 was also more prevalent in ADC (5.8%) than in SCC (3.4%) (*P=*0.04).

**Figure 2:**
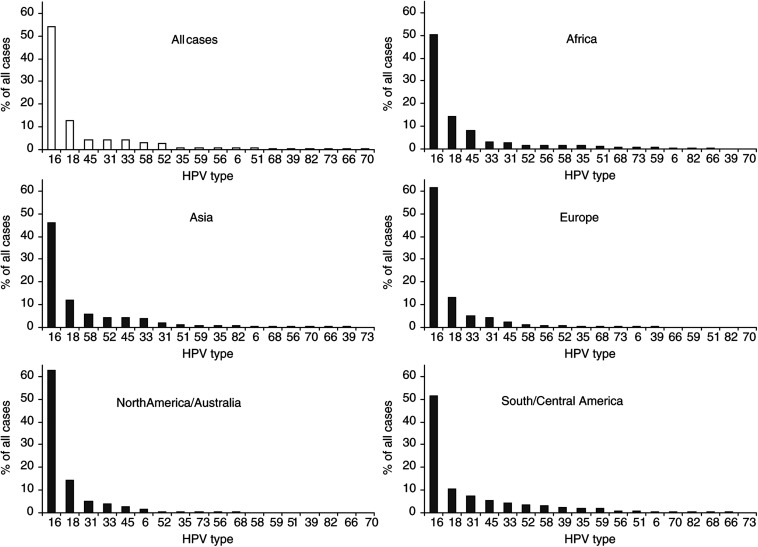
Type-specific prevalence of HPV in 8550 cases of squamous cell and unspecified cervical carcinoma by region.

Comparison of HPV-specific prevalence in SCC by region is shown in Figure 2

. In SCC, HPV16 was the predominant type in all regions studied, varying from 45.9% in Asia to 62.6% in North America and Australia. HPV18 was found consistently in 10–14% of SCC cases. In most regions, HPV45 (2–8%), 31 (2–7%) and 33 (3–5%) were the most prevalent types in SCC after types 16 and 18. In cases from Africa, the prevalence of HPV45 (8.0%) was more than twice that of either 31 (2.7%) or 33 (3.2%). In cases from Asia, HPV58 (5.8%) and 52 (4.4%) were found more commonly than HPV45, 31 and 33. Other HPV types varied considerably in their prevalence from region to region, but accounted for no more than 2% of ICC cases from any region.

**Figure 3:**
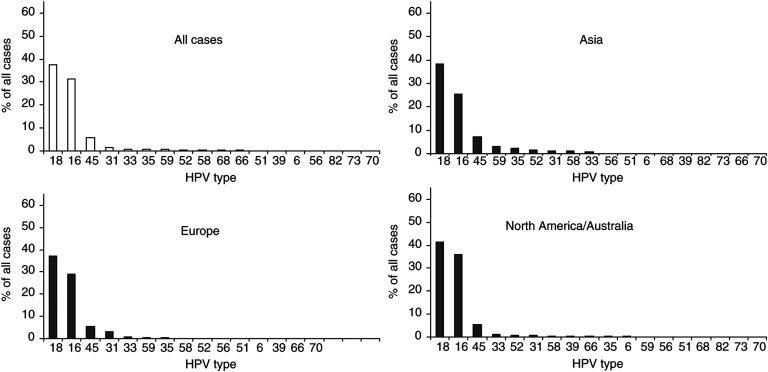
Type-specific prevalence of HPV in 1508 cases of adeno- and adenosquamous cervical carcinoma by region.

Sufficient ADC-specific data existed for the comparison of HPV-specific prevalence across Asia, Europe and North America and Australia (Figure 3

). HPV18 was the predominant type (37.7%), found consistently in 37–41% of ADC cases in these regions, with HPV16 accounting for a smaller proportion (26–36%). HPV45 was the third most prevalent in each region, present in 5–7% of ADC cases *vs* only in 2–4% of SCC cases from these regions. The HPV16 phylogenetically related types 31, 33, 52 and 58 (but not 35) were all less prevalent in ADC cases than in SCC cases from each region.

## Discussion

Two-thirds of ICC cases included in this meta-analysis were associated with HPV16 (51.0%) or 18 (16.2%) infection. However, more than 16 other HPV types were also associated with ICC, of which the most prevalent were types 45, 31, 33, 58 and 52 (collectively accounting for 18.3% of cases). The HPV16 family of viruses were more commonly found in SCC than ADC, whereas the HPV18 family were more common in ADC.

### Geographical region

Overall detected HPV prevalence varied little between geographical regions (83–89%), but was low compared to the almost 100% HPV prevalence identified in studies using the most sensitive HPV detection methods (Walboomers *et al*, 1999). This reflects the fact that many studies used HPV DNA detection strategies of suboptimal sensitivity. When comparing prevalence by region and histology, we attempted to take account of alternative HPV DNA sources and PCR primers by adjustment. However, it is not known to what extent other unknown sources of variation such as sample storage conditions, specific PCR conditions and quality of histopathology may affect these comparisons. Residual differences in prevalence between regions could also be because of the yet unknown HPV types not amplified by the existing PCR primers.

There were many similarities in HPV type-specific distribution across the regions studied. In SCC, HPV16 was clearly the predominant type varying from 45.9% in Asia to 62.6% in Europe, with HPV18 being found consistently in 10–14% of the cases. Other rarer types appeared to vary in their distribution. In most regions, HPV45, 31 and 33 were the third, fourth and fifth most common genotypes, although not necessarily in that order. Asia appeared to be different with a larger proportion of cancers associated with HPV58 and 52, as highlighted by a recent study in China of 786 cases in which HPV58 and 52 were the third (10%) and fourth (9%) most common genotypes in ICC (Wong *et al*, 2000). Other types in SCC were too rare to make inferences on region-specific variations.

### Histological type of ICC

This meta-analysis shows that overall HPV prevalence detected in ADC was significantly lower than that detected in SCC. This intriguing finding does not appear to be because of differences with respect to region or HPV detection methods as it persisted even after adjusting for these factors. ADC arises from tissue deeper in the interior of the cervix uteri than SCC, and it has been reported to be more difficult to appropriately sample exfoliated cells of ADC than SCC (Sasieni and Adams, 2001). However, most HPV detected in the present review was based on biopsy specimens (77%). A proportion of cervical ADC could be misclassified ADC arising from the endometrium or other rare histological variants of ADC, for example, clear cell and mesonephric, which have been suggested to be HPV-independent (O'Leary *et al*, 1998; Pirog *et al*, 2000).

Whereas HPV16 was the most common type in SCC followed by HPV18, the situation was reversed in ADC where HPV18 was the most common type, followed closely by HPV16. This difference has been described independently by many of the studies in this analysis and by studies outside the scope of this review (IARC, 1995). Compared to HPV16, HPV18 has been shown to be associated with increasing oncogenic potential in cell culture (Barbosa and Schlegel, 1989), as well as a more rapid transition to malignancy (Burger *et al*, 1996) and a poorer prognosis of cancer patients (Nakagawa *et al*, 1996; Hildesheim *et al*, 1999; Schwartz *et al*, 2001). Given the fact that columnar tissue giving rise to ADC is less accessible, and possibly less susceptible to HPV infections, than the squamous tissue of SCC, the establishment of ADC may require a relatively more aggressive infection. In addition to HPV16 and 18, this large meta-analysis facilitated the identification of differences for some rarer phylogenetically related types: the HPV16-related types 31, 33, 35, 52, and 58 were more prevalent in SCC (15.0% collectively) than in ADC (4.4% collectively); and HPV18-related 45 was more prevalent in ADC (5.8%) than SCC (4.2%). All these differences were seen consistently in all regions where the comparison was possible.

For all regions where histological comparison was possible, the ratio of ADC to SCC was higher than that reported by cancer registries (Parkin *et al*, 1997). For example, ADC represent 23.1% of histologically verified cases from Europe in this study, but only 15.3% of ICC cases reported to European cancer registries (Parkin *et al*, 1997). Since all seven studies of only ADC cases were from Europe, Japan or USA, ADC is over-represented in this meta-analysis, particularly in developed countries. No material differences in results were observed when SCC was compared with cancers of unspecified histology.

### Study limitations

The different PCR primers employed by the studies covered in this analysis varied in their overall detection of HPV DNA, with the highest prevalence being obtained with SPF10 and GP5+/6+ primers, supporting findings from previous studies (Davies *et al*, 2001). Such variation is partly because of known differences in the range of HPV DNA types amplifiable by each primer set, and this was taken into consideration when estimating type-specific prevalence. However, there is also evidence that not all primer sets amplify individual HPV types with the same sensitivity (Qu *et al*, 1997; Kleter *et al*, 1999), and such differences are a potential source of bias in this analysis.

The type-specific prevalence reported for each individual type includes that in multiple infections, which were reported in a total of 3.7% of our ICC cases. Since many of the included studies tested for only a subset of HPV types, many multiple infections will have been missed. Hence, this meta-analysis was unable to estimate how often individual HPV types were found in the presence of other types, which limits the conclusions that can be made about individual HPV-type oncogenicity. In particular, a large proportion of cases positive for HPV6, which is not thought to be oncogenic, may be coinfected with an undetected HR HPV type.

The cases included in this meta-analysis are not representative of the worldwide distribution of ICC. The proportion of cases contributed by Africa (6.1%) and Asia (30.7%) in this study under-represent their proportional burdens of worldwide cervical cancer, which are 14.1 and 49.4%, respectively (Parkin *et al*, 1999). In contrast, the proportion of cases contributed by Europe (33.2%) and North America and Australia (15.5%) over-represent their proportional burdens, which are 15.7 and 4.4%, respectively. Adjustment of type-specific prevalence in all ICC cases (Figure 1) by weighting each region according to their cancer burden, however, did not materially effect the results, highlighting the general similarity of HPV-type distribution across regions (data not shown).

Furthermore, the cases in this meta-analysis were not drawn uniformly from across each region. Large areas have not been included (e.g., the Middle East and Indian subcontinent in Asia), while other specific populations such as Japan in Asia are over-represented. There is also evidence of inter-regional variation in HPV-type distribution; the high prevalence of HPV52 and 58 in Asia is more apparent in cases from China/Korea/Japan than in those from South East Asia. Hence, for the comparison of alternative regional groupings, HPV-specific prevalence is presented by study in the Appendix (Table A1).

Given that HPV is considered a virtually necessary cause of ICC, we further examined results restricted to HPV DNA-positive cases. This increased type-specific prevalence by a factor of ≈1.1 consistently for each HPV type, with no impact on the relative distribution of HPV types. However, given that many of the PCR systems used by the included studies amplify only a subset of HPV types, many HPV-‘negative’ cases may actually be infected with other, unascertained, HPV types. Thus, we did not consider it appropriate to restrict to HPV-positive cases when comparing type-specific prevalence across studies where PCR methodology differed considerably.

However, in order to make a broad overall estimate if one does assume that, HPV DNA should be detectable in 100% of ICC and that the distribution of undetected types in HPV-negative cases is similar to that in positive cases, this meta-analysis suggests that vaccinating against HPV16 and 18 should prevent over 70% of worldwide ICC. However, a worldwide vaccine against only HPV16/18 may prevent a larger proportion of ICC in Europe, North America and Australia (≈75%), than in Africa, Central and South America and Asia (59–64%), where a larger proportion of ICC cases were associated with other HPV types. Although this study identifies at least 18 HPV types associated with ICC from around the world, the most important type after HPV16 and 18 appears to be HPV45, followed by types 31, 33, 58 and 52, the relative importances of which vary by region.

## Appendix A

A comparison of alternative regional groupings, HPV prevalence is presented by the study in Table AI

**Table AI:** Study methods and prevalence of human papillomavirus by study and by region

							**HPV prevalence (% of all cases tested)**																		
**First author**	**Reference**	**Country**	**HPV DNA source**	**PCR primers used to identify all HPV +ve**	**No. cases**	**SCC (incl. unspec)/ADC**	**Any**	**16**	**18**	**45**	**31**	**33**	**58**	**52**	**35**	**59**	**56**	**51**	**6**	**68**	**39**	**82**	**73**	**66**	**70**
*Africa*																									
Bosch FX	*JNCI* (1995)	Algeria, Benin, Guinea, Mali,Uganda,Tanzania	Fresh biopsies	My09/11	186	186/0	89.8	42.5	17.7	12.4	2.7	2.7	2.7	2.2	2.2	0.0	3.2	1.1	0.0	2.2		0.5		0.0	
Bayo S	*Int J Epidemiol* (2002)	Mali	Fresh biopsies	GP5+/6+	65	65/0	96.9	47.7	12.3	10.8	0.0	1.5	3.1	0.0	1.5	0.0	0.0	6.2	0.0	0.0	0.0		3.1	0.0	
Chaouki N	*Int J Cancer* (1998)	Morocco	Exfol. cells	GP5+/6+	186	173/13	94.6	67.7	12.4	4.8	3.8	2.7	0.0	1.1	0.5	1.6	0.5	0.0	0.5	0.0	0.0	0.0	0.0	0.5	0.0
Lin P	*Cancer Epid Biomark Prev* (2001)	Senegal	Exfol. cells	MY09/11 +HMB01	51	51/0	64.7	37.3	7.8	9.8	0.0	5.9	2.0	3.9	2.0	0.0	2.0	0.0	2.0	0.0	0.0		0.0	0.0	
Williamson AL	*J Med Virol* (1994)	South Africa	Fresh biopsies	MY09/11	68	60/8	80.9	45.6	1.5	1.5	5.9	5.9							0.0						
ter Meulen J	*Int J Cancer* (1992)	Tanzania	Exfol. cells	GP 5/6	53	53/0	88.7	37.7	32.1	5.7	0.0	1.9													
**Region subtotal**					**609**	**588/21**	**88.8**	**50.2**	**14.1**	**7.9**	**2.6**	**3.1**	**1.6**	**1.6**	**1.4**	**0.6**	**1.6**	**1.2**	**0.4**	**0.8**	**0.0**	**0.3**	**0.7**	**0.2**	**0.0**
																									
*Asia*																									
Huang S	*Int J Cancer* (1997)	China	Fresh biopsies	MY09/11	40	35/5	87.5	27.5	30.0	0.0	0.0	0.0	27.5	27.5	0.0	0.0	0.0	0.0	0.0	0.0	0.0	0.0		0.0	0.0
Lin QQ	*Int J Cancer* (1998)	China	Fresh/fixed biopsies	MY09/11, GP5+/6+	77	77/0	93.5	48.1	5.2	1.3	2.6	3.9	18.2	5.2	0.0	2.6	0.0	0.0	10.4	0.0	0.0	0.0	0.0	0.0	1.3
Lo KWK	*Gynecol Obstet Invest* (2001)	China	Fresh biopsies	MY09/11	121	107/14	78.5	48.8	11.6	0.0	0.8	5.0	6.6	0.8	0.0	0.0	0.8	0.0	0.0	0.0	0.0		0.0	0.0	0.0
Peng H	*Int J Cancer* (1991)	China	Exfol. cells	TS-PCR only	101	92/9	34.7	31.7				3.0													
Stephen AL	*Int J Cancer* (2000)	China	Fixed biopsies	GP5+/6+ of TS-PCR neg samples only	34	24/10	88.2	61.8	8.8	2.9	0.0	2.9	2.9	0.0	0.0	2.9	0.0	0.0	0.0	0.0	0.0		0.0	0.0	
Munirajan AK	*Gynecol Oncol* (1998)	India	Fresh biopsies	pU-1M/pU-2R	43	43/0	69.8	53.5	9.3		0.0	2.3	2.3	0.0	2.3	0.0	0.0		0.0						
Bosch FX	*JNCI* (1995)	Indonesia, Philippines, Thailand	Fresh biopsies	My09/11	98	98/0	96.9	42.9	31.6	8.2	1.0	2.0	2.0	2.0	1.0	1.0	3.1	0.0	0.0	1.0		4.1		0.0	
Fujinaga Y	*J Gen Virol* (1991)	Japan	Fresh biopsies	pU-1M/pU-2R	39	39/0	84.6	48.7	12.8		5.1	5.1	7.7	2.6					0.0						
Harima Y	*Int J Radiat Oncol Biol Phys* (2002)	Japan	Fresh biopsies	pU-1M/pU-2R	84	79/5	76.2	26.2	4.8		2.4	2.4	7.1	2.4	0.0	0.0	0.0		1.2						
Kashiwabara K	*Acta Pathol Japan* (1992)	Japan	Fixed biopsies	L1C1/C2	93	68/25	58.1	48.4	6.5		0.0	1.1	0.0	3.2					0.0						
Maki H	*Jpn J Cancer Res* (1991)	Japan	Biopsies	L1 PCR	29	29/0	82.8	44.8	20.7			6.9	6.9	6.9					0.0						
Nagai Y	*Am J Clin Oncol* (2001)	Japan	Exfol. cells	L1C1/C2	293	239/54	85.3	25.3	3.1		3.8	4.1	3.1		1.7										
Nakagawa S	*Cancer* (1996)	Japan	Fresh biopsies	L1C1/C2 +C2M	146	116/30	88.4	37.7	18.5	0.7	2.1	6.2	8.2	10.3	2.1	0.0	0.0	0.7	0.0	2.1	0.0			0.0	0.7
Nawa A	Cancer (1995)	Japan	Fresh/fixed biopsies	E6C1/C2	23	23/0	87.0	73.9	13.0			0.0													
Saito J	*Gynecol Obstet Invest* (2000)	Japan	Fixed biopsies	pU-1M/pU-2R	66	66/0	75.8	34.8	12.1			10.6	1.5	6.1											
Sasagawa T	*Cancer EpidBiomark Prev* (2001)	Japan	Exfol. cells	LCR-E7	84	72/12	89.3	42.9	14.3	1.2	6.0	1.2	3.6	10.7	1.2	0.0	0.0	4.8	1.2	0.0	0.0		0.0	0.0	0.0
Yamakawa Y	*Gynecol Oncol* (1994)	Japan	Fixed biopsies	TS-PCR only	64	0/64	67.2	32.8	39.1		1.6	0.0			7.8				0.0						
Hwang T	*J Korean Med Sci* (1999)	Korea	Exfol. cells	pU-1M/pU-2R	41	39/2	92.7	36.6	9.8		7.3	9.8	17.1	7.3	2.4										
Kim KH	*Yonsei Med J* (1995)	Korea	Fixed biopsies	WD72/76 + WD66/67/154	30	30/0	70.0	53.3	16.7		0.0	0.0													
Yadav M	*Med J Malaysia* (1995)	Malaysia	Fresh biopsies	MY09/11	23	23/0	95.7	73.9	65.2		13.0	4.3													
Ngelangel C	*JNCI* (1998)	Philippines	Fresh biopsies+exfol. cells	GP5+/6+	356	323/33	93.5	38.8	25.6	12.9	0.6	0.0	2.5	2.8	0.0	2.0	0.6	2.5	0.0	0.6	0.3	0.3	0.3	1.1	0.0
Chen SL	*Cancer* (1993)	Taiwan	Fresh biopsies	L1C1/C2, E6C1/C2/C3	43	40/3	72.1	46.5	4.7		0.0	2.3	2.3	2.3					0.0						
Chen TM	*Int J Cancer* (1994)	Taiwan	Fresh biopsies	MY09/11, pU-1M/pU-2R	433	382/51	79.0	46.2	12.2			6.5							0.0						
Lai HC	*Int J Cancer* (1999)	Taiwan	Fresh biopsies	MY09/11	94	87/7	86.2	47.9	8.5	0.0	2.1	4.3	18.1	2.1	0.0	0.0	0.0	0.0	0.0	1.1	0.0	0.0	0.0	0.0	0.0
Yang YC	*Gynecol Oncol* (1997)	Taiwan	Fixed biopsies	MY09/11 of TS-PCR neg samples only	136	120/16	83.1	63.2	6.6		0.7	5.1													
Bhattarakosol P	*J Med Assoc Thai* (1996)	Thailand	Fixed biopsies	MY09/11	100	100/0	82.0	35.0	17.0			3.0							0.0						
Chichareon S	*JNCI* (1998)	Thailand	Fresh biopsies+exfol. cells	GP5+/6+	377	338/39	94.7	54.4	20.7	1.6	1.9	1.3	2.7	2.4	0.5	1.6	0.0	0.0	0.5	0.0	0.5	0.0	0.0	0.0	0.5
Siritantikorn S	*Southeast Asian J Trop Med Public Health* (1997)	Thailand	Lavage	My09/11	23	21/2	60.9	56.5	4.3		4.3	4.3			0.0				0.0						
**Region subtotal**					**3091**	**2710/381**	**83.1**	**43.4**	**15.3**	**4.5**	**2.0**	**3.4**	**5.4**	**4.2**	**1.0**	**1.1**	**0.4**	**1.0**	**0.5**	**0.5**	**0.2**	**0.4**	**0.1**	**0.3**	**0.3**
																									
*Europe*																									
Birner P	*Mod Pathol* (2001)	Austria	Fixed biopsies	GP 5+/6+	86	86/0	88.4	68.6	8.1	7.0	8.1	41.9	2.3	0.0	1.2	0.0	0.0	0.0	0.0	0.0	0.0	0.0	1.2	0.0	0.0
Baay MFD	*J Clin Microbiol* (2001)	Belgium	Fixed biopsies	GP5+/6+ of TS-PCR neg samples only	115	95/20	87.8	67.0	13.0	1.7	2.6	1.7	0.0	0.0	0.0	0.0	0.9	0.0	0.0	0.0	0.0	0.0	0.0	0.9	0.0
Tachezy R	*J Med Virol* (1999)	Czech Republic	Fresh biopsies	MY09/11	49	49/0	73.5	59.2	10.2	2.0	4.1	0.0	2.0	0.0	0.0	0.0	0.0	0.0		0.0	4.1	0.0	0.0	0.0	0.0
Hording U	*APMIS* (1997)	Denmark	Fixed biopsies	TS-PCR only	50	0/50	70.0	18.0	52.0			0.0													
Sebbelov AM	*Microbes Infect* (2000)	Denmark	Fixed biopsies	GP5/6 of TS-PCR neg samples only	34	34/0	85.3	70.6	0.0	0.0	0.0	5.9			0.0										
Iwasawa A	*Cancer* (1996)	Finland	Fixed biopsies	MY09/11	460	352/108	88.0	63.5	25.0			2.6												0.0	
Lombard I	*J Clin Oncol* (1998)	France	Fresh biopsies	TS-PCR of SBH neg samples only	297	269/28	82.8	50.5	10.1	0.3	1.0	2.0	1.0	0.3	0.3				0.0		0.0				
Riou G	*Lancet* (1990)	France	Biopsies	TS-PCR of SBH neg samples only	106	89/17	84.0	54.7	16.0			5.7							0.0						
Milde-Langosch K	*Int J Cancer* (1995)	Germany	Biopsies	MY09/11	51	25/26	80.4	51.0	25.5		3.9	0.0			0.0				0.0						
Nindl I	*International Papillomavirus Conference Proceedings* (2001)	Germany	Fixed biopsies	My09/11	77	77/0	89.6	42.0	16.0		18.0	6.0	0.0	1.0	1.0			0.0	8.0		1.0			1.0	
Bosch FX	*JNCI* (1995)	Germany, Poland, Spain	Fresh biopsies	MY09/11	86	86/0	95.3	65.1	8.1	2.3	5.8	1.2	1.2	3.5	1.2	0.0	2.3	0.0	0.0	3.5	0.0	0.0		1.0	
Dokianakis DN	*Oncol Rep* (1999)	Greece	Pap smears	GP 5/6	77	75/2	58.4	2.6	36.4			3.9													
Koffa M	*Int J Oncol* (1994)	Greece	Fixed biopsies	GP 5/6	39	32/7	76.9	35.9	38.5			10.3													
Labropoulou V	*Sex Transmis Dis* (1997)	Greece	Fresh biopsies	MY09/11	35	35/0	97.1	54.3	22.9	0.0	5.7	0.0	0.0	0.0	0.0	0.0	0.0	0.0	0.0	0.0	0.0	0.0		0.0	
Sebbelov AM	*Microbes Infect* (2000)	Greenland	Fixed biopsies	GP5/6 of TS-PCR neg samples only	32	32/0	84.4	81.3	0.0	0.0	3.1	3.1			0.0										
Konya J	*J Med Virol* (1995)	Hungary	Fresh biopsies	L1C1/C2 +C2M	47	41/6	97.9	55.3	40.4		2.1	0.0	0.0	2.1					0.0						
O'Leary JJ	*J Clin Pathol* (1998)	Ireland	Fixed biopsies	GP5/6, GP1/2	20	20/0	90.0	80.0	10.0			0.0							0.0						
Sjyldberg BM	*Mod Pathol* (1999)	Ireland, Sweden	Fixed biopsies	GP 5+/6+	38	0/38	60.5	23.7	26.3		0.0	0.0													
Garzetti GG	*Cancer* (1998)	Italy	Fresh biopsies	GP 5/6	32	32/0	68.8	50.0	15.6		6.3	0.0			0.0				0.0						
Voglino G	*Pathologica* (2000)	Italy	Fixed biopsies	MY09/11, pU1M/2R	145	120/25	98.6	71.0	6.9		16.6	2.1			0.6										
Karlsen F	*J Clin Microbiol* (1996)	Norway	Fresh biopsies	My09/11, GP5+6+, Oli1b/2I, CpI/II	361	361/0	98.3	68.4	14.1		1.1	8.3			0.6										
Kleter B	*J Clin Microbiol* (1999)	Russia	Fixed biopsies	SPF10	180	129/51	100.0	64.4	9.4	7.8	3.9	1.1	1.7	1.1	1.7	0.0	2.2	0.0	0.0	0.0	0.0	0.0	0.0	0.0	0.0
Munoz N	*Int J Cancer* (1992)	Spain	Exfol. cells	MY09/11	142	142/0	69.0	45.8	3.5		3.5	3.5			1.4				0.0		0.0			0.0	
Rodriguez JA	*Diag Mol Pathol* (1998)	Spain	Fixed biopsies	GP 5+/6+	54	54/0	85.2	61.1	7.4	5.6	5.6	1.9	1.9	0.0	0.0	0.0	0.0	0.0	0.0	0.0	0.0	0.0	0.0	0.0	0.0
Andersson S	*Eur J Cancer* (2001)	Sweden	Fixed biopsies	My09/11	173	0/173	68.2	23.9	36.4	5.2	0.6	0.0	0.0	0.0	0.0	0.6	0.0	0.0	0.0		0.0				
Hagmar B	*Med Oncol Tumor Pharmacother* (1992)	Sweden	Fixed biopsies	MY09/11	71	71/0	74.6	38.0	9.9		16.9	8.5							0.0						
Wallin KL	NEJM (1999)	Sweden	Fixed biopsies	MY09/11, GP 5+/6+	104	85/19	76.9	47.1	25.0	0.0	2.9	5.8	0.0	0.0	0.0	0.0	0.0	0.0	0.0	0.0	0.0	0.0	1.0	0.0	0.0
Zehbe I	*J Pathol* (1997)	Sweden	Fixed biopsies	GP 5+/6+	45	38/7	95.6	53.3	20.0	6.7	0.0	13.3	2.2	0.0	0.0		0.0	0.0	0.0						
Baay MFD	*Eur J Gynaec Oncol* (1996)	The Netherlands	Fixed biopsies	MY09/11, GP5/6, CpI/II	162	162/0	87.7	61.7	14.2	0.6	1.9	4.3	0.6	0.0	0.0	0.0	0.0	0.0	0.0	0.0	0.0	0.0		0.0	0.0
van den Brule AJC	*Int J Cancer* (1991)	The Netherlands	Fresh biopsies	GP5/6 + GP1/2	50	50/0	100.0	84.0	26.0		4.0	2.0							0.0						
Arends MJ	*Hum Pathol* (1993)	UK	Fixed biopsies	TS-PCR only	47	26/21	78.7	53.2	29.8			0.0							0.0						
Crook T	*Lancet* (1992)	UK	Fresh biopsies	TS-PCR of SBH neg samples only	28	23/5	89.3	71.4	17.9		0.0	0.0							0.0						
Giannudis A	*Int J Cancer* (1999)	UK	Fixed biopsies	GP 5+/6+	43	43/0	100.0	81.4	9.3	2.3	0.0	4.7	0.0	0.0	0.0	0.0	0.0	0.0	0.0	0.0	2.3			0.0	
**Region subtotal**					**3336**	**2733/603**	**85.9**	**56.0**	**17.5**	**2.9**	**4.2**	**4.4**	**0.8**	**0.5**	**0.5**	**0.1**	**0.6**	**0.0**	**0.3**	**0.4**	**0.3**	**0.0**	**0.3**	**0.1**	**0.0**
																									
*North America and Australia*																									
Chen S	*Int J Gynecol Obstet* (1999)	Australia	Fresh biopsies	My09/11	186	153/33	91.9	53.8	17.2	4.8															
Thompson CH	*Gynecol Oncol* (1994)	Australia	Fresh/fixed biopsies	pU-1M/pU-2R	103	103/0	86.4	65.0	18.4		2.9	0.0	0.0												
Duggan MA	*Human Pathol* (1995)	Canada	Fixed biopsies	L1C1/C2 of DBH neg samples only	76	0/76	69.7	35.6	39.5		0.0	2.6			0.0				0.0						0.0
Bosch FX	*JNCI* (1995)	USA, Canada	Fresh biopsies	My09/11	57	57/0	93.0	57.9	15.8	14.0	5.3	0.0	0.0	0.0	0.0	0.0	3.5	0.0	0.0	1.8		0.0		0.0	
Burger RA	*JNCI* (1996)	USA	Fresh biopsies	My09/11	401	297/104	84.8	51.4	19.7	2.2	2.5	1.0	0.5	1.0	0.0	0.0	0.0	0.0	0.5	0.2	0.0	0.0	0.2	0.0	0.0
Burnett AF	*Gynecol Oncol* (1992)	USA	Fixed biopsies	My09/11	21	18/3	100.0	71.4	28.6		42.9	4.8			19.0				0.0						
Ferguson AW	*Mod Pathol* (1998)	USA	Fixed biopsies	MY09/11	27	0/27	59.3	25.9	25.9	7.4															
Paquette RL	*Cancer* (1993)	USA	Fresh biopsies	MY09/11	45	28/17	93.3	48.9	40.0		4.4								0.0						
Schwartz SM	*J Clin Oncol* (2001)	USA	Fixed biopsies	MY09/11	465	354/111	88.2	58.5	23.2	1.3	2.4	3.4	0.0	0.0	0.2	0.0	0.0	0.0	2.4	0.0	0.2	0.0	0.2	0.2	0.0
Sebbelov AM	*Microbes Infect* (2000)	USA	Fixed biopsies	GP5/6 of TS-PCR neg samples only	53	53/0	98.1	77.4	3.8	0.0	20.8	30.2			0.0										
Wistuba II	*Cancer Res* (1997)	USA	Fixed biopsies	SPF10	20	20/0	90.0	60.0	20.0		5.0	5.0													
Resnick RM	*JNCI* (1990)	USA (+Holland)	Fixed biopsies	My09/11, WD72/76	33	29/4	100.0	75.8	15.2	3.0	3.0	3.0							0.0						
Pirog EC	*Am J Pathol* (2000)	USA (+Poland)	Fixed biopsies	SPF10	76	0/75	89.5	39.5	38.2	10.5	1.3	1.3	0.0	2.7	0.0	0.0	0.0	0.0	0.0	0.0	0.0			0.0	
**Region subtotal**					**1562**	**1112/450**	**87.5**	**54.9**	**22.1**	**3.3**	**3.9**	**3.2**	**0.2**	**0.6**	**0.4**	**0.0**	**0.2**	**0.0**	**1.1**	**0.2**	**0.1**	**0.0**	**0.2**	**0.1**	**0.0**
																									
*South and Central America*																									
Bosch FX	*JNCI* (1995)	Argentina,Bolivia,Brazil,Chile,Colombia,Cuba,Panama,Paraguay	Fresh biopsies	MY09/11	505	505/0	92.9	50.5	9.5	7.3	6.9	3.6	2.2	3.2	2.0	2.8	0.6	1.0	0.2	0.4		0.2			
Alonio LV	*MEDICINA* (2000)	Argentina	Biopsies	GP 5+/6+	30	30/0	93.3	46.7	20.0		3.3	3.3							6.7						
Eluf-Neto J	*Br J Cancer* (1994)	Brazil	Exfol. cells	GP 5/6	186	186/0	84.4	53.8	9.7		2.2	3.2							0.0						
Lorenzato F	*Int J Gynecol Cancer* (2000)	Brazil	Exfol. cells	MY09/11	59	59/0	89.8	59.3	38.5	0.0	11.9	5.1	3.4	1.7	3.4				0.0						
Rabelo-Santos SH	*International Papillomavirus Conference Proceedings* (2000)	Brazil	Fixed biopsies	GP 5+/6+	56	51/5	80.4	57.1	5.4		1.8	8.9	0.0	0.0		0.0		0.0	0.0						
Munoz N	*Int J Cancer* (1992)	Colombia	Exfol. cells	MY09/11	87	87/0	72.4	50.6	5.7		5.7	0.0			3.4				0.0						
Herrero R	*JNCI* (2000)	Costa Rica	Exfol. cells	MY09/11 +HMB01	34	34/0	88.0	47.0	15.0	0.0	5.9	8.8	12.0	0.0	2.9	2.9	2.9	2.9	2.9	5.9	0.0		0.0	2.9	0.0
Ferrera A	*Int J Cancer* (1999)	Honduras	Exfol. cells	MY09/11	104	99/5	80.6	47.1	13.5	5.8	3.8	9.6	6.7	1.0	0.0	1.0	0.0	0.0	1.0	0.0	0.0	0.0	0.0	0.0	0.0
Torroella-Kouri M	*Gynecol Oncol* (1998)	Mexico	Fresh biopsies+Exfol. Cells	MY09/11 +HMB01	69	59/10	87.0	42.0	14.5	10.1	2.9	0.0	4.3	2.9	0.0	4.3	1.4	0.0	0.0	1.4	4.3	1.4	0.0	1.4	1.4
Meyer T	*J Infect Dis* (1998)	Mexico	Fresh biopsies	MY09/11	60	60/0	96.7	43.3	16.7	3.3	23.3	6.7	0.0	1.7	1.7	0.0	1.7	0.0	3.3	0.0	1.7	3.3	0.0	0.0	0.0
Illades-Aguiar B	*International Papillomavirus Conference Proceedings* (2001)	Mexico	Fresh biopsies	MY09/11	74	66/8	97.3	60.8	10.8	0.0	10.8	1.4	4.1	2.7	0.0	0.0	0.0	0.0	1.4	0.0	0.0	0.0	0.0	0.0	
Santos C	*Br J Cancer* (2001)	Peru	Fresh biopsies	GP5+/6+	196	171/25	94.9	56.1	12.2	4.6	9.7	4.1	2.0	7.7	3.6	0.5	1.0	1.0	0.0	0.0	2.6	0.0	0.0	1.0	0.5
Lo KWK	*Int J Cancer* (2002)	China	Fresh biopsies	MY09/11, GP5+/6+	809	731/78	83.7	66.9	6.3	0.4	0.6	1.5	3.2	2.2											
Mortazavi SH	*Asian Pacific J Cancer Prev* (2002)	Iran	Fixed biopsies	TS-PCR only	69	61/8	85.5	73.9	11.6			1.2													
																									
Ishikawa H	*Cancer* (2001)	Japan	Fixed biopsies	TS-PCR only	52	52/0	76.9	53.8	23.1			1.9													
Dybikowska A	*Oncol Rep* (2002)	Poland	Exfol. cells	MY09/11	53	53/0	53.8	48.1	0.0		1.9	1.9	1.9						0.0						
Pegoraro RJ	*Int J Gynecol Cancer* (2002)	South Africa	Fresh biopsies	MY09/11	190	190/0	98.4	46.8	14.2	4.7		10	1.1	1.6	1.6			0.0	0.5						
Cuzick J	*Br J Cancer* (2000)	UK	Exfol. cells	MY09/11	116	85/31	94	65.5	11.2	0.0	11.2	6.9	1.7	0.0	1.7	0.0	1.7	0.0	0.0	0.0	0.0	0.0	0.0	0.0	0.0
**Region subtotal**					**1460**	**1407/53**	**89.3**	**51.7**	**10.6**	**5.5**	**7.0**	**4.0**	**2.9**	**3.3**	**2.0**	**1.9**	**0.7**	**0.7**	**0.5**	**0.5**	**1.7**	**0.4**	**0.0**	**0.4**	**0.4**
																									
**Total**				**10 058**	**8550/2725**	**86.0**	**51.0**	**16.3**	**4.4**	**3.9**	**3.8**	**2.7**	**2.3**	**0.9**	**0.8**	**0.6**	**0.5**	**0.6**	**0.5**	**0.4**	**0.2**	**0.2**	**0.2**	**0.2**	
